# Risk Factors for Problematic Behaviors among Forensic Outpatients under the Medical Treatment and Supervision Act in Japan

**DOI:** 10.3389/fpsyt.2016.00144

**Published:** 2016-08-24

**Authors:** Kumiko Ando, Takahiro Soshi, Kanako Nakazawa, Takamasa Noda, Takayuki Okada

**Affiliations:** ^1^Department of Forensic Psychiatry, National Center of Neurology and Psychiatry, National Institute of Mental Health, Tokyo, Japan; ^2^Department of Psychiatry, National Center of Neurology and Psychiatry, National Center Hospital, Tokyo, Japan; ^3^Section of Psychiatry and Behavioral Science, Tokyo Medical and Dental University Graduate School, Bunkyo-ku, Tokyo, Japan

**Keywords:** medical treatment and supervision act, forensic outpatient, outpatient treatment, problematic behavior, risk factor

## Abstract

The Medical Treatment and Supervision Act (MTSA) was enacted in 2005 in Japan to promote the reintegration of clinical offenders with mental disorders into society. Under the MTSA, individuals who committed serious crimes in a state of insanity or diminished responsibility are diverted from the criminal justice system to the mental health system. Based on court decisions about MTSA-based treatment, clinical offenders have an obligation to engage in rehabilitation within their local community under the guidance of mental health professionals. However, patients under MTSA-based clinical treatments have faced various problems in the course of treatment, because of psychiatric as well as other static or dynamic factors, and sometimes have committed problematic behaviors, such as violence and medical non-compliance. Hence, this study aimed to clarify factors related to patients’ inclusion in MTSA-based outpatient treatment and additionally, their commitment of problematic behaviors, based on confidential data acquired during a four-year government survey period (National Center of Neurology and Psychiatry) from MTSA enactment (July 15, 2005) to December 31, 2009. In total, we recruited 441 clinical offenders receiving MTSA-based outpatient treatment from 158 nationwide facilities. To evaluate related factors, we collected demographic, psychiatric, forensic, clinical treatment, and social service information. Statistical analyses demonstrated that predominant profiles of patients included male gender, younger age, low school history, psychiatric diagnoses (F1, F2, and F3), and no correctional or outpatient history before MTSA-based treatment. F1 or substance use diagnosis, in particular, was increasingly correlated with other factors, such as male gender, older age, and correctional history before MTSA treatment. Among the 441 patients, 189 (43%) committed problematic behaviors in the course of the MTSA-based outpatient treatment. Risk factors for patients’ commitment of problematic behaviors comprised F1 diagnosis and inpatient history before MTSA-based treatment inclusion. In summary, reduction of overall problematic behaviors under the MTSA outpatient likely makes progress by focal attention to patients with psychiatric disorders caused by substance use and/or a past inpatient history for more severe psychiatric symptoms. This work is of ongoing and future importance in the domain of forensic community treatment, to connect risk-enhancing factors with risk management.

## Introduction

Until the recent enactment of the Medical Treatment and Supervision Act (MTSA) in 15 July 2005 (1), neither legislation nor facilities for offenders with mental disorders were available in Japan (see the following site for recent information of the MTSA by the Ministry of Health, Labour, and Welfare, Japan: http://www.mhlw.go.jp/stf/seisakunitsuite/bunya/hukushi_kaigo/shougaishahukushi/sinsin/index.html). The aims of the MTSA are to improve symptoms of clinical offenders, who are defined as patients committing criminal acts because of their mental health status, to prevent them from re-offending and to promote their rehabilitation into local communities by providing them with adequate support systems for continuous and appropriate medical care.

**Figure 1:**
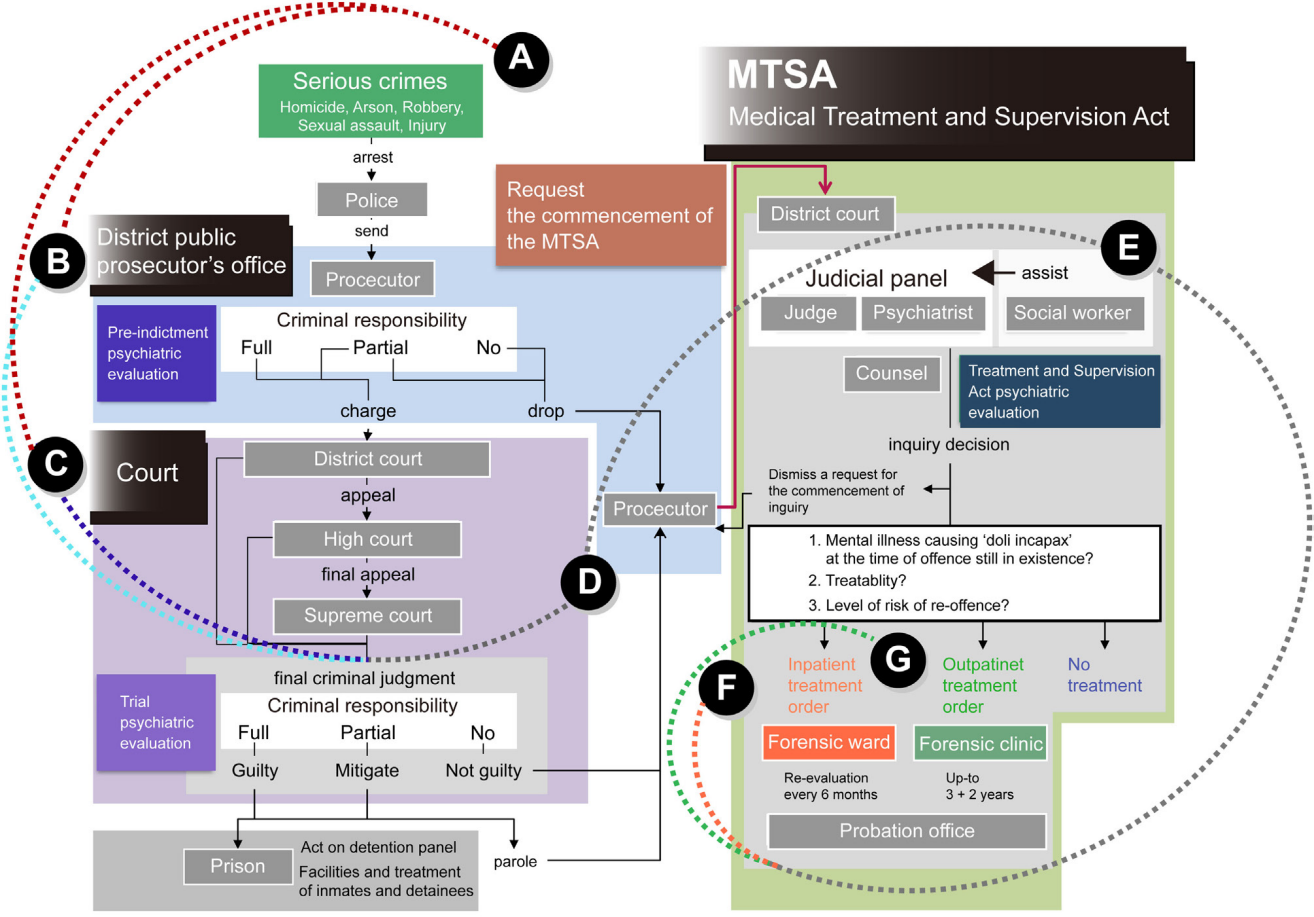
**Flow of the Medical Treatment and Supervision Act (MTSA) in Japan**. The MTSA is applied to clinical offending populations who commit **(A)** “serious crimes,” such as homicide, arson, robbery, sexual assault, and injury under the condition of insanity or diminished responsibility. Clinical offenders are exempted from **(B)** prosecution, which is managed at district public “prosecutor offices,” or are provided with **(C)** a suspended sentence on the grounds of their insanity or diminished capacity at “Court”; patients are consequently found not guilty, and are given a reduced sentence. After **(D)** a prosecutor makes allegation concerning medical treatment and supervision under the “MTSA,” **(E)** a “judicial panel” is formed to determine the requirements of medical treatment orders and treatment contents. The panel consists of a judge and a psychiatrist as a forensic mental health specialist, which are supported by a social worker. When requirements of medical treatment of patients under the MTSA are recognized by a verdict determined by a judicial panel, patients undergo **(F)** an “inpatient treatment” or **(G)** an “outpatient treatment” at designated medical facilities.

Treatment flow pertaining to the MTSA is summarized in Figure 1. The MTSA is applicable to specific offending populations with mental disorders who commit serious crimes, such as homicide, arson, robbery, rape/sexual assaults (henceforth, sexual assault), and physical assaults causing bodily injury resulting in death (injury) under the condition of insanity or diminished responsibility (“Serious crime”; Figure 1A). Because they are exempt from prosecution (“District public prosecutor office“; Figure 1B) or given a suspended sentence on the grounds of insanity or diminished capacity (“Court”; Figure 1C), they are found not guilty but given a reduced sentence. After a prosecutor makes allegations concerning medical treatment and supervision under the MTSA (“Prosecutor”; Figure 1D), a judicial panel, which consists of a judge and psychiatrist as a publically licensed forensic mental health specialist, supported by a social worker, is formed to determine the medical treatment orders and treatment content (“Judicial panel”; Figure 1E). When medical treatment of offenders is required under the MTSA, based on a verdict of the judicial panel, offenders are placed mainly under the supervision of probation facilities, and undergo inpatient treatment in a designated medical institution (“Inpatient treatment order”; Figure 1F), or outpatient treatment at a designated outpatient medical institution (“Outpatient treatment order”; Figure 1G).

For inpatient treatment, a range of treatment programs appropriate to the patient’s disorder and coping ability are offered by a multidisciplinary team comprised of psychiatrists, nurses, psychiatric social workers, clinical psychologists, and occupational therapists, in one of the MTSA-designated hospitals throughout Japan. There is no time limit for hospitalization; however, guidelines for inpatient treatment suggest a period of approximately 18 months. In hospitals, treatment is separated into acute, recovery, and rehabilitation phases.

When the court’s decision specifies outpatient treatment, this is also provided by a multidisciplinary team at one of the designated outpatient facilities across the country. Specifically, treatment is given in three divided stages (early, middle, and late) following an outpatient treatment schedule developed by the probation office. In principle, the duration of outpatient treatment is to be 3 years, up to a maximum of 5 years. In addition, during outpatient treatment, a rehabilitation coordinator from the probation office, in collaboration with the local mental health authority, conducts mental health monitoring. This involves regular interviews with the patient, and monitoring to ensure the continuous provision of medical care and support to promote reintegration of the individual into society. According to a report by the Ministry of Health, Labor and Welfare, there were 368 designated outpatient facilities in Japan as of July 2010, and 799 offenders had received MTSA-based outpatient treatment as of July 2010 (2, 3) (for the latest public information, also visit to the site: http://www.mhlw.go.jp/stf/seisakunitsuite/bunya/hukushi_kaigo/shougaishahukushi/sinsin/index.html).

It is expected that MTSA-based medical treatment may promote reintegration of clinical offenders into society, and lead to increased overall social well-being and prevention of their re-offence. However, various risk factors exist that may cause conflicting or problematic behaviors in supervised forensic psychiatric contexts. These factors may not only hinder smooth rehabilitation but also have the potential to trigger re-offending of patients. Such risk factors belong to two main categories (4). Static risk factors include male gender, younger age, genetic factors, family environment in the past (e.g., antisocial parental behavioral style), and offending history (4–10). Dynamic risk factors include interpersonal conflict, personality, impulsivity, psychiatric symptoms, and substance use (4, 9, 11–13).

Because such risk factors have been reported in previous cohort studies from other countries, it may also be important for Japan to obtain information about whether such risk factors are similarly significant in the domestic forensic psychiatric context, and what kinds of factors are associated with problematic behaviors during MTSA-based treatments. Doing so may promote the rehabilitation of clinical offenders into local communities, as well as establish overall social well-being and safety. In particular, it may be necessary to establish an assessment methodology or guideline to prospectively evaluate potential patients included in MTSA treatment and/or with the potential for future problematic behaviors to prevent various negative public effects, such as socio-economic loss (14, 15).

The aim of this study, therefore, was to identify factors related to clinical offenders’ inclusion in MTSA-based outpatient treatment, and risks for their engagement in problematic behaviors, using data from a four-year survey of the National Center of Neurology and Psychiatry (NCNP) from 15 July 2005 to 31 December 2009. A categorical analysis was employed to elucidate correlations between problematic behaviors and other characteristics of patients, such as demographics, psychiatric status, forensic history, clinical treatment, and social service use.

## Materials and Methods

### Participants

We indirectly recruited clinical offenders receiving outpatient treatment under the MTSA at cooperating designated outpatient facilities by contacting clinical staffs through postal mail or e-mail. Among a total of 368 nationwide facilities, the 158 designated facilities obtained patients’ voluntary consensuses and provided us their agreement to cooperate with the study. First, we included 461 clinical offenders who had undergone MTSA-based outpatient treatment as of 31 December 2009, or the year before the 2010 survey. After correcting and merging data from patients who had received treatment from multiple facilities because of transfers, 441 patients were chosen for inclusion in this study. Hence, it is estimated that we were able to collect data on 55% of the overall population (*N* = 799). The 441 patients were included into MTSA-based outpatient treatment either directly (*n* = 207; 46.9%) or indirectly (*n* = 234; 53.1%) after being hospitalized for psychiatric assessments over a period of several months (16, 17) during the period from 15 July 2005 to 31 December 2009. A verdict about direct outpatient treatment order or others is presented by a judicial parallel established within the District Court, based on the criterions of patients’ mental illness, treatability, and risks for re-offending (Figure 1E) (1).

### Procedure

The self-making survey sheets were sent by mail to designated outpatient facilities every year from 2005, or the year of the MTSA enactment, to follow changes in patients’ treatment and living circumstances, such as their treatment facilities and employment. The survey sheet consists of various questions about demographic, psychiatric, forensic, and clinical-related items. Question items were not directly answered by clinical offenders, but rather by clinical staff in designated facilities. About 1 month after delivery of survey sheets, completed data sheets were collected from the facilities by surface mails. Research assistants confirmed that all required fields were completed. If missing answers could not be elucidated or extracted from other responses, research assistants asked again clinical staffs in outpatient facilities to help with omitted answers by phone or e-mail. Other research assistants also checked and corrected incorrect inputs. Data filled with completed responses were finally stored on a standalone personal computer located inside a locked administrative room. In follow-up or subsequent years, new survey sheets, which also included information from previous survey years, were sent again to outpatient facilities to collect patients’ latest information as well as to obtain re-confirmation of previous data from clinical staff.

### Survey Items

Overall, data about patients receiving MTSA outpatient treatment were collected in five main categories: demographics, psychiatric status, forensic background, clinical treatment history, and social service utilization. We indirectly collected the patients’ data from clinical staffs at designated facilities. Therefore, collected data tend to include static factors, such as age, sex, but not dynamic factors, such as personality and impulsivity traits, which can be collected by direct measure of patients’ self-assessments or interviews.

Demographic information was collected regarding age, sex, and school history (junior high or less, high school, or university). Personal information, such as full names, personal addresses, and phone numbers was not collected to prevent identification of individuals. Employment and marital status factors were not included, because almost all patients were unemployed (about 90%), and their employments might change between before and after the supervised outpatient treatment. Additionally, many patients were unmarried, and the marital status complicatedly interacts with the residence status: the unmarried status is not always equal to solitude, which may strongly affect treatment success. Marital and residence problems are generally ongoing social problems in not only forensic psychiatric domains but also an overall aging society. Hence, we may independently examine additional data from cooperative facilities from a broader perspective.

Psychiatric information was obtained based on the official nosological system of the 10th revision of the International Statistical Classification of Diseases and Related Health Problems (ICD-10). The presence or absence of the following diagnoses was indicated by responding “yes” or “no”: F0, organic and symptomatic mental disorders; F1, mental and behavioral disorders due to psychoactive and other substance use; F2, schizophrenia, schizotypal, and delusional disorders; F3, mood or affective disorders; F4, neurotic, stress-related, and somatoform disorders; F5, behavioral syndromes and mental disorders with physiological dysfunction; F6, adult personality and behavior disorders; F7, mental retardation; F8, psychological development disorders; and F9, behavioral and emotional disorders.

Upon F1 diagnosis, users of substances, such as illegal drugs, get arrested not necessarily for criminal acts under drug influence, but possession of drugs tends to be a crime globally. Hence, patients with F1 diagnosis are potentially criminal according to a control law, whether they committed criminal acts. F1 patients under MTSA-based outpatient treatment, however, committed criminal acts under severe mental disorders, and hence, are distinguished from those with only use and possession of illegal substances. Probably, F1 patients under MTSA-based treatment have already possessed severe symptoms of mental disorders, which likely cause constant substance use and criminal behaviors. Therefore, we included F1 patients similarly with patients with other diagnoses, although their mental states and problematic behaviors may not be clearly separated from non-psychiatric or organic factors.

Criminal or forensic information included past admission to correctional institutions, type of criminal offense for the MTSA-based treatment decision (homicide, arson, robbery, sexual assault, and injury), and victim type (familiar, including family members, patients themselves or acquaintances, or strangers). History of correctional admission was summarized as a binary response of “Yes” or “No.”

Clinical treatment information consisted of outpatient pathway (direct or indirect transfer), past outpatient (yes or no), past inpatient treatment (medical care or involuntary inpatient, or voluntary inpatient) before the MTSA-based treatment, and frequency of outpatient visits per month at the start of MTSA-based treatment (one or more visits, one visit, or no visits). Inpatient history was summarized as “Yes” or “No.”

Social service information included frequency of day care use per week at the start of MTSA-based treatment (one or more times, one time, or no use).

### Statistical Analysis

We counted the number of patients in each category for each information or variable, and tested observed frequencies with chi-square tests, which examined dominant profiles related to patients’ MTSA-based treatment inclusion and their problematic behaviors in a stepwise manner. A chi-square test was used to calculate expected frequencies (EF) in each cell in cross tables (EF_*i*,*j*_ = ROF*_i_* × COF*_j_*/*N*; EF = expected frequency; ROF = observed frequencies in rows; COF = observed frequencies in columns; *i* = number of rows; *j* = number of columns; *N* = total number of samples). A χ^2^ score was produced by the following equation: χ^2^ = Σ[(OF − EF)^2^/EF]; OF = observed frequency in each cell. It was tested with a χ^2^ distribution [*Y* = (1/2)*^k^*^/2^/γ(*k*/2) × *x^k^*^/2−1^ × *e*^−^*^x^*^/2^; *k* = degree of freedoms; *Γ* = a gamma function; *x* = χ^2^ scores], for a given number of degrees of freedom [*df* = (*i* − 1) × (*j* − 1)]. To detect factors related to problematic behaviors as sensitively as possible, and also to avoid Type II or conservative errors, we did not control significance levels for multiple comparisons. Statistical analyses were performed using IBM SPSS Statistics for Windows OS, version 19 (IBM Corp., Tokyo, Japan).

Only three dominant diagnoses were included for the psychiatric variable, F1 (*n* = 26), F2 (*n* = 340), and F3 (*n* = 43), because the others included too few patients for statistical analysis. In addition, sexual assaults and injury were merged into the single category “assault” for the type of criminal offense variable.

First, we examined the types of variables that were predominantly related to patients’ inclusion in MTSA-based treatment. Although we included frequencies of MTSA-based outpatient treatment and day care in this analysis, these factors were related to clinical conditions after the MTSA decision, and not prospective factors affecting the MTSA decision *per se*. Consequently, even if statistically significant effects were found, these MTSA-based treatments were not counted as a related factor. Victim type was also discarded from prospective consideration, because it does not prospectively affect engagement in offenses for MTSA decisions, but is related to an aspect of offending acts. We also tested correlations between psychiatric diagnoses and other variables based on chi-square tests with odd ratios (OR), which are described below.

Second, we aimed to elucidate which variables were related to problematic behaviors. We counted the number of patients with or without problematic behaviors for each of the demographic, psychiatric, forensic, clinical, and social service variables, and examined the relationships between problematic behaviors and other variables with chi-square tests. Although we counted numbers of sub-types of problematic behaviors, the present study examined relations between overall problematic behaviors and other profiles of patients. Odds ratios were produced for significant variables to examine strengths and directions of correlations between problematic behaviors and variables, using the following equation: OR = [RF_(+)_ in PB_(+)_/RF_(−)_ in PB_(+)_]/[RF_(+)_ in PB_(−)_/RF_(−)_ in PB_(−)_]; RF_(+)_ = positive response (e.g., “Yes” to F2 diagnosis); RF_(−)_ = negative response (e.g., “No” to F2 diagnosis); PB_(+)_ = presence of problematic behaviors; PB_(−)_ = absence of problematic behaviors. In addition, 95% confidence intervals of ORs were also calculated with the following equation: 95% CI = exp[log(OR) ± 1.96 × sqrt(Σ(1/OF_i_))]; sqrt = square root; OF = observed frequencies; *i* = cell numbers in a cross table. When significant interactions were found, we reported both observed and expected frequencies in each cell, and also, ORs and 95% CIs to comprehend interaction properties. Interaction effects among significant variables were also tested using chi-square tests. Odd ratios and 95% CIs were calculated manually.

### Ethical Considerations

The present study was carried out in accordance with the recommendations of the Ethical Guidelines for Epidemiological Research of the Japanese Ministry of Education, Culture, Sports, Science and Technology and the Ministry of Health, Labor, and Welfare. The research protocol was approved by the Ethics Committee of the National Center of Neurology and Psychiatry (NCNP) in accordance with the Declaration of Helsinki. Patients gave informed consent to clinical staffs in cooperating designated facilities, and clinical staffs provided us their facility agreements to cooperate with the study. Personal names, addresses, and phone numbers of patients were never included in surveys, to maintain anonymity in accordance with the ethical criterion of the NCNP human clinical research protocol.

## Results

### MTSA-Based Outpatient Treatment

Demographic, clinical, forensic, clinical treatment, and social service profiles of the 441 patients are represented in Table 1. The predominant profiles of patients were the following: (i) male gender; (ii) younger age; (iii) low school history; (iv) F1, F2, and F3 diagnoses; (v) no correctional history; and (vi) outpatient history before the MTSA-based treatment.

**Table 1:** **Demographic, psychiatric, forensic, clinical treatment, and social service profiles of patients receiving MTSA-based outpatient treatment (*n* = 441)**.

Variable		*n* (%) or mean ± SD
Sex		
	Women	131 (30)
	Men	310 (70)
Age (years)		43.6 ± 12.9
	20s	65 (15)
	30s	132 (30)
	40s	102 (23)
	50s	82 (19)
	≥60s	60 (14)
Education		
	Junior high or less	167 (38)
	High school	187 (42)
	University/college	87 (20)
Psychiatric diagnosis (ICD-10)		
	F0	5 (1)
	F1	26 (6)
	F2	340 (77)
	F3	43 (10)
	F4	5 (1)
	F5	0 (0)
	F6	5 (1)
	F7	7 (2)
	F8	6 (1)
	F9	0 (0)
Crime type		
	Homicide	109 (25)
	Arson	148 (32)
	Robbery	20 (5)
	Sexual assault	29 (7)
	Injury (physical assault)	142 (32)
Victim type		
	Familiar	275 (62)
	Stranger	176 (38)
History of admission to correctional institutions		32 (7)
Outpatient pathway (direct/indirect)		207/234
Outpatient history (yes)		344 (78)
Inpatient history (yes)		236 (54)
Frequency of outpatient visits (one month)		
	No visits	63 (14)
	One visit	11 (2)
	>One visit	367(83)
Frequency of day care use (1 week)		
	No use	234 (53)
	One time	53 (12)
	>One time	154(35)
Problematic behaviors (yes)		189 (43)

*F0: organic and symptomatic mental disorders; F1: mental and behavioral disorders due to psychoactive and other substance use; F2: schizophrenia, schizotypal, and delusional disorders; F3: mood or affective disorders; F4: neurotic, stress-related, and somatoform disorders; F5: behavioral syndromes and mental disorders associated with physiological dysfunction; F6: disorders of adult personality and behavior; F7: mental retardation; F8: disorders of psychological development; F9: behavioral and emotional disorders with onset usually occurring in childhood or adolescence. Others (n = 4) include diagnoses, such as G40 (epilepsy), which do not appear here*.

#### Demographic Profiles

The patients consisted of 310 men (70.3%) and 131 women (29.7%) [$\chi_{\left(1\right)}^{2}$ = 72.655, *p* < 0.0001]. The proportion of female clinical offenders was slightly higher than that of general offenders (about 20% at the time of this survey). This trend seems to reflect the fact that female patients suffering from mood disorders (F3), such as depression, are likely to become patients under the MTSA, as will be reported later. The mean age was 43.6 ± 12.9 years old. The proportion of patients in their 30s and 40s (*n* = 234) was larger than those in older generations[$\chi_{\left(4\right)}^{2}$ = 39.465, *p* < 0.0001]. Lower school history was also prevalent [the combined proportion for junior high or less (*n* = 167) or high school (*n* = 187) was greater than for university (*n* = 87); $\chi_{2}^{2}$ = 38.095, *p* < 0.0001].

#### Psychiatric Profiles (ICD-10 Codes)

**Figure 2:**
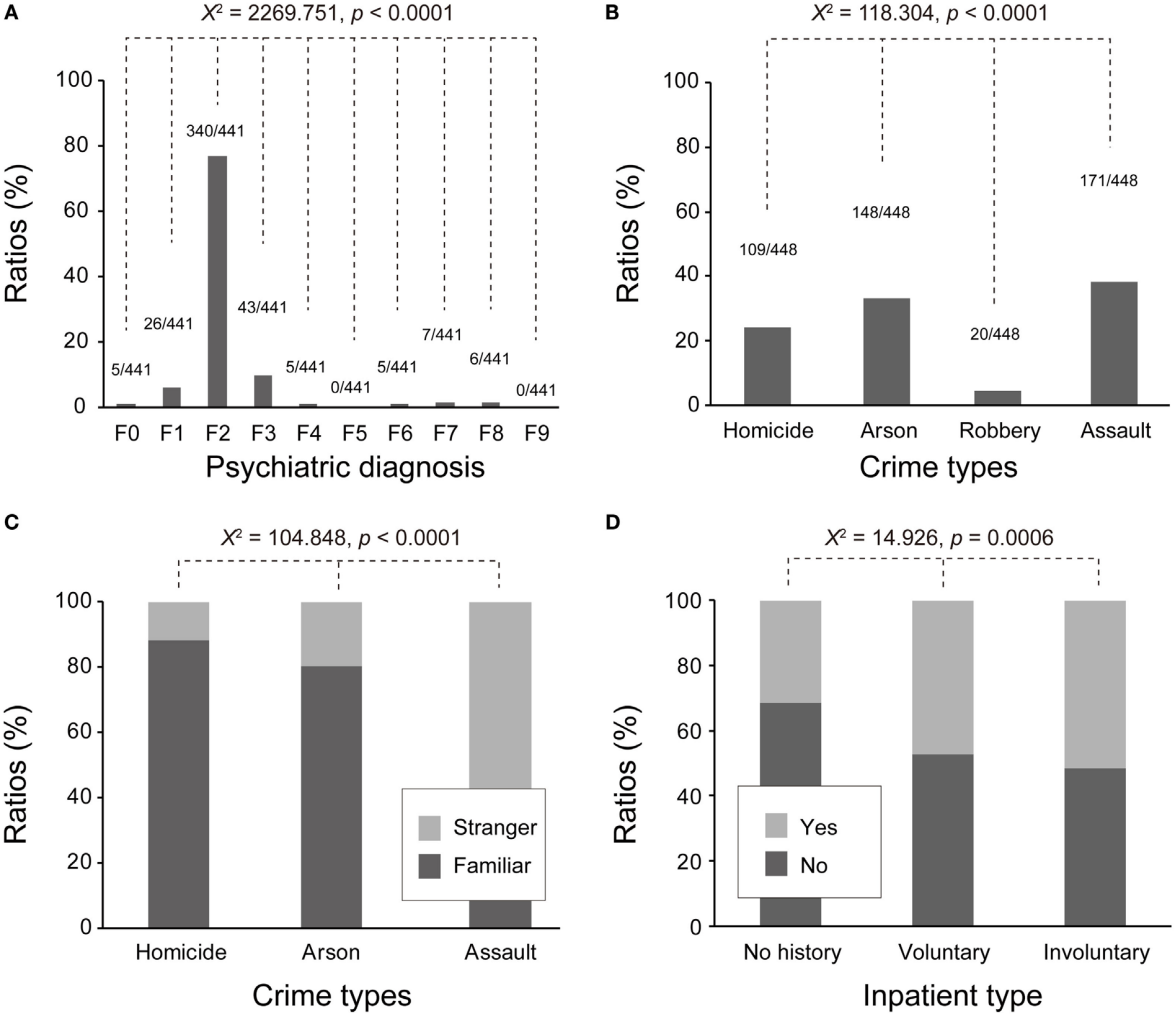
**Summary of psychiatric, forensic, and clinical (inpatient history) profiles of patients (*n* = 441) under MTSA-based outpatient treatment (15 July 2005 to 31 December 2009)**. **(A)** Psychiatric disorders were diagnosed with ICD-10 (F0–F9), as described in detail in the Section “Materials and Methods.” Predominant psychiatric diagnoses were F2 (77.0%, *n* = 340), F3 (10.0%, *n* = 43), and F1 (6.0%, *n* = 26). **(B)** Patients committed four dominant types of crimes, including assaults (39.0%, *n* = 171), arson (34.0%, *n* = 148), homicide (25.0%, *n* = 109), and robbery (5.0%, *n* = 20). Assault consists of sexual assault and injury. **(C)** The dominant crime types were different in terms of the proportion of victim types: homicide and arson mainly involved familiar victims (homicide: 88.1%; arson: 80.4%). On the other hand, assault victims mainly were strangers (64.3%). **(D)** Among patients with voluntary inpatient history (*n* = 118), about half (52.5%, *n* = 62) did not commit any problematic behaviors; however, another half (47.5%; *n* = 56) did. Among patients with involuntary inpatient history, 48.4% (*n* = 76) did not commit problematic behaviors, while 51.6% (*n* = 81) did. Both voluntary and involuntary inpatient histories were risk factors for problematic behaviors. Involuntary inpatient history showed a higher odds ratio (OR = 2.337) than voluntary inpatient history (OR = 1.980).

The frequencies of psychiatric diagnoses (F0–F9) were significantly different [$\chi_{9}^{2}$ = 2269.751, *p* < 0.0001] (Figure 2A). The most prevalent psychiatric diagnosis was schizophrenia (F2), which accounted for 77.0% (*n* = 340). The second most prevalent was mood or affective disorders (F3: 10.0%, *n* = 43), and third was mental and behavioral disorders due to psychoactive substance use (F1: 6.0%, *n* = 26). These dominant psychiatric disorders were introduced throughout subsequent analyses.

#### Forensic Profiles under the MTSA

The most common offense was assault which includes sexual assault and injury, with 171 cases (39.0%), followed by arson (34.0%, *n* = 148), homicide (25.0%, *n* = 109), and robbery (5.0%, *n* = 20). The frequencies of these offenses were significantly different [$\chi_{\left(3\right)}^{2}$ = 118.304, *p* < 0.0001] (Figure 2B).

With respect to crime victims, familiar people accounted for 62.0% (*n* = 275) and strangers or others 38.0% (*n* = 176). A cross-tabulation of relationships between offenses and victims indicated that a large proportion of victims of homicide and arson were familiar people (88.1% and 80.4%, respectively). On the other hand, assault victims were mainly strangers (64.3%). The proportions of victim types for the three dominant crimes were significantly different [$\chi_{\left(2\right)}^{2}$ = 104.848, *p* < 0.0001] (Figure 2C).

#### History of Admission to Correctional Institutions before MTSA Treatment

Before the offense related to MTSA treatment, 32 patients (7.0%) had been admitted to correctional institutions, while most (93.0%, *n* = 409) had never been admitted [$\chi_{\left(1\right)}^{2}$ = 322.288, *p* < 0.0001].

#### MTSA-Based Outpatient Treatment Profiles

The pathways to outpatient treatment after hospitalization for assessment include (i) direct outpatient treatment, which starts under the MTSA after an initial ruling specifying medical care without hospitalization; and (ii) indirect or transfer treatment, in which patients are initially treated at a designated hospital, and then, move to outpatient treatment. About half of the patients (*n* = 207, 46.9%) underwent direct treatment, and 234 patients (53.1%) received indirect treatment, and these frequencies were not significantly different [$\chi_{\left(1\right)}^{2}$ = 1.653, *p* = 0.199]. The frequencies for outpatient treatment per month at the start of MTSA-based treatment were significantly different [$\chi_{\left(2\right)}^{2}$ = 503.075, *p* < 0.0001], and those who had been outpatients more than once accounted for a significantly larger proportion than other frequencies.

#### History of Psychiatric Treatment before MTSA-Based Treatment

In total, 344 patients (78.0%) had received previous outpatient treatment, and almost all had received treatment before the offense. This proportion of patients was significantly larger than the proportion without the treatment [$\chi_{\left(1\right)}^{2}$ = 138.342, *p* < 0.0001]. On the other hand, with respect to history of inpatient treatment prior to the MTSA decision, 236 patients (54.0%) had undergone treatment, a proportion that was not significantly different from those without inpatient history [$\chi_{\left(1\right)}^{2}$ = 2.179, *p* = 0.140]. Among the total number of 375 cases, 77 (32.6%) had experienced involuntary admission, 127 (53.8%) underwent hospitalization for medical care and protection, and 117 (49.6%) were administered under voluntary admission. Manner of admission was not known for the remaining patients (22.9%; *n* = 54). The distribution of admission types showed significant differences [$\chi_{\left(3\right)}^{2}$ = 37.405, *p* < 0.0001].

#### MTSA-Based Social Service Profiles

The frequencies of day care use per week at the start of MTSA treatment were significantly different [$\chi_{\left(2\right)}^{2}$ = 111.932, *p* < 0.0001]. Patients without use (*n* = 234) or use on more than one occasion (*n* = 154) accounted for a larger proportion than those with only one use (*n* = 53).

#### Relationships between Psychiatric Diagnoses and Other Variables

To clarify relationships between dominant psychiatric diagnoses (F1, F2, and F3) and other factors, we performed chi-square tests. The results summarized in Table 2 demonstrate that an F1 diagnosis was widely related to various profiles.

**Table 2:** **Direction and strength of significant correlations (odd ratios) between the three dominant psychiatric diagnoses and each variable**.

	F1		F2		F3	
Variable	OR	95% CI	OR	95% CI	OR	95% CI
Sex (ref. women)						
Men	3.419	1.009–11.595			0.36	0.190–0.681
Age (ref. 20s)						
30s	0.733	0.119–4.496				
40s	2.681	0.551–13.043				
50s	2.046	0.384–10.903				
≥60s	4.846	0.986–23.826				
School history (ref. ≤junior high)						
High school	3.048	1.267–7.333				
University/college	4.384	1.698–11.320				
Crime type (ref. no count)						
Homicide						
Arson					0.451	0.216–0.940
Assault					2.175	1.131–4.184
Correctional history (ref. no admission)	13.759	5.633–33.625	0.228	0.109–33.475		
Outpatient history (ref. no history)	0.424	0.186–0.968				
Outpatient pathway (ref. indirect)	0.372	0.158–0.874	2.98	1.865–4.762	0.27	0.132–0.550
Inpatient history (ref. no history)						

*F1: mental and behavioral disorders due to psychoactive and other substance use; F2: schizophrenia, schizotypal, and delusional disorders; F3: mood or affective disorders; ref: reference or baseline for comparison; OR: odds ratio; 95% CI: 95% confidence interval*.

Gender was significantly related to F1 and F3, but not F2 diagnoses [F1: $\chi_{\left(1\right)}^{2}$ = 4.367, *p* = 0.045; F3: $\chi_{\left(1\right)}^{2}$ = 10.506, *p* = 0.002; F2: $\chi_{\left(1\right)}^{2}$ = 1.536, *p* = 0.264]. Men (7.4%), compared to women (2.3%), were proportionally more often diagnosed with F1. Conversely, F3 yielded a reverse pattern (men/women: 7/17%).

Age was significantly related to F1 diagnosis [$\chi_{\left(4\right)}^{2}$ = 10.739, *p* = 0.028]. Compared to patients in their 20s (3%) as reference, those 40s and older had proportionally more F1 diagnoses [30s (2%), 40s (8%), 50s (6%), 60s and older (13%)]; however, all 95% CI ranges included the ratio score of one, which casts doubt about the significance of the ORs. Other diagnoses were not significantly related to age [F2: $\chi_{\left(4\right)}^{2}$ = 2.976, *p* = 0.505; F3: $\chi_{\left(4\right)}^{2}$ = 7.083, *p* = 0.130].

School history was significantly related to F3 diagnosis [F3: $\chi_{\left(2\right)}^{2}$ = 10.702, *p* = 0.004; F1: $\chi_{\left(2\right)}^{2}$ = 5.304, *p* = 0.067; F2: $\chi_{\left(2\right)}^{2}$ = 1.353, *p* = 0.516]. Compared to those with junior high or less, patients with higher education were more often diagnosed with F3.

Next, we examined relationships between crime types and psychiatric diagnoses. Homicide was significantly related to F3 diagnosis [F3: $\chi_{\left(1\right)}^{2}$ = 5.623, *p* = 0.018; F1: $\chi_{\left(1\right)}^{2}$ = 0.040, *p* = 0.842; F2: $\chi_{\left(1\right)}^{2}$ = 0.286, *p* = 0.593]. Patients committing homicide were more frequently diagnosed with F3 (40%). Assault was also significantly related to F3 diagnosis [F3: $\chi_{\left(1\right)}^{2}$ = 4.704, *p* = 0.030; F1: $\chi_{\left(1\right)}^{2}$ = 1.529, *p* = 0.216; F2: $\chi_{\left(1\right)}^{2}$ = 2.607, *p* = 0.106]. However, patients committing assaults were diagnosed less frequently with F3. Arson was not significantly related to any diagnosis [F1: $\chi_{\left(1\right)}^{2}$ = 0.097, *p* = 0.756; F2: $\chi_{\left(1\right)}^{2}$ = 3.783, *p* = 0.052; F3: $\chi_{\left(1\right)}^{2}$ = 0.037, *p* = 0.847].

Correctional history before the MTSA-based treatment was associated with F1 and F2 diagnoses [F1: $\chi_{\left(1\right)}^{2}$ = 50.441, *p* < 0.0001; F2: $\chi_{\left(1\right)}^{2}$ = 17.849, *p* < 0.0001; F3: $\chi_{\left(1\right)}^{2}$ = 0.006, *p* = 0.941]. Patients with correctional history were more frequently diagnosed with F1; however, F2 showed a reverse pattern.

Outpatient history before the MTSA-based treatment was significantly related to F1 diagnosis [F1: $\chi_{\left(1\right)}^{2}$ = 4.366, *p* = 0.037; F2: $\chi_{\left(1\right)}^{2}$ = 0.580, *p* = 0.446; F3: $\chi_{\left(1\right)}^{2}$ = 2.985, *p* = 0.084]. Patients with outpatient history were diagnosed less often with F1. The outpatient pathway was also significantly associated with the three dominant diagnoses [F1: $\chi_{\left(1\right)}^{2}$ = 5.513, *p* = 0.019; F2: $\chi_{\left(1\right)}^{2}$ = 21.863, *p* < 0.0001; F3: $\chi_{\left(1\right)}^{2}$ = 14.446, *p* < 0.0001]. Patients receiving direct outpatient treatment were more often diagnosed with F2; however, F1 and F3 demonstrated reverse patterns.

Inpatient history was not significantly correlated with the three dominant diagnoses [F1: $\chi_{\left(1\right)}^{2}$ = 0.137, *p* = 0.711; F2: $\chi_{\left(1\right)}^{2}$ = 1.316, *p* = 0.251; F3: $\chi_{\left(1\right)}^{2}$ ≈ 0.000, *p* = 0.997].

### Risk Factors for Problematic Behaviors under MTSA-Based Outpatient Treatment

Among the 441 patients receiving MTSA-based treatment, 189 (42.9%) committed problematic behaviors. A total of 293 problematic behaviors were counted, which included dominant problematic behaviors, such as medical non-compliance (25%), violent behaviors (24%), substance abuse (10%), and self-harming and suicide (7%). The present study examined the relationship between the summarized problematic behaviors and each factor. We conducted chi-square tests, and calculated ORs for significant factors. The results are summarized in Table 3. Two significant relationships were found. F1 diagnosis was significantly associated with problematic behaviors [$\chi_{\left(1\right)}^{2}$ = 7.847, *p* = 0.007]. F1 diagnosis, compared to no diagnosis, demonstrated a higher risk for problematic behaviors.

**Table 3:** **Summary of patient profiles with (*n* = 189) or without (*n* = 252) problematic behaviors under MTSA-based outpatient treatment**.

Variable	Levels	Problematic behaviors, *n* (EF) or mean ± SD				
		No	Yes	χ2	*p*-value	OR (95% CI)
Sex	Men	170	140	2.262	0.141	
	Women	82	49			
Age (years)		44 ± 13	43 ± 13	0.554^a^	0.580	
	20s	33	32	2.135	0.711	
	30s	80	52			
	40s	56	46			
	50s	47	35			
	≥60s	36	24			
School history	Junior high or less	91	76	1.342	0.505	
	High school	107	80			
	University/college	54	33			
Psychiatric diagnosis						
F1	No (ref.)	244 (237)	171 (178)	7.847	0.007**	3.211 (1.365–7.554)
	Yes	8 (15)	18 (11)			
F2	No	50	51	3.121	0.086	
	Yes	202	138			
F3	No	225	173	0.621	0.517	
	Yes	27	16			
Outpatient pathway	Direct	119	88	0.019	0.923	
	Indirect	133	101			
Outpatient history	No	62	35	2.330	0.133	
	Yes	190	154			
Inpatient history	No (ref.)	128 (117)	77 (88)	4.388	0.043*	1.502 (1.026–2.198)
	Yes	124 (135)	112 (101)			
History of correction	No	239	170	3.844	0.063	
	Yes	13	19			
Crime type						
Homicide	No	183	149	2.243	0.148	
	Yes	69	40			
Arson	No	165	128	0.245	0.684	
	Yes	87	61			
Robbery	No	242	179	0.436	0.645	
	Yes	10	10			
Assault	No	163	108	2.592	0.114	
	Yes	89	81			
Victim type	Stranger	87	79	2.435	0.136	
	Familiar	165	110			
Frequency of outpatient visits (one month)	No visits	39	24	1.230	0.559	
	One visit	5	6			
	>One visit	208	159			
Frequency of day care use (1 week)	No use	138	98	4.794	0.091	
	One time	36	17			
	>One time	78	74			

*EF, expected frequencies of patients in a chi-square test; OR, odds ratio (yes/no); 95% CI, 95% confidence interval; ref, reference for comparison*.

*^a^*t*-value; F1: mental and behavioral disorders due to psychoactive and other substance use; F2: schizophrenia, schizotypal, and delusional disorders; F3: mood (affective) disorders. Assault consists of sexual assault and injury*.

*p < 0.05.

**p < 0.01.

Additionally, inpatient history before MTSA-based treatment yielded a similar effect [$\chi_{\left(1\right)}^{2}$ = 4.388, *p* = 0.043]. Inpatient history, compared to no history, was related to a higher risk for problematic behaviors. To test relations between types of inpatient histories and problematic behaviors, we further examined in detail whether patients received care voluntarily or involuntarily (medical care or involuntary admission) likely because voluntary or involuntary inpatient history provides information about the severity of patients’ mental disorders. As represented in Figure 2D, among patients with voluntary inpatient history (*n* = 118), 52.5% (*n* = 62) did not commit any problematic behaviors, while 47.5% (*n* = 56) did. On the other hand, among patients with involuntary inpatient history, 48.4% (*n* = 76) did not commit problematic behaviors, while 51.6% (*n* = 81) did. A chi-square test with two levels of problematic behavior (yes or no) and three levels of inpatient type (no, voluntary, and involuntary) showed a significant effect [$\chi_{\left(2\right)}^{2}$ = 14.926, *p* = 0.0006]. Both voluntary and involuntary inpatient histories, compared to no history, were risk-enhancing factors for problematic behaviors, and involuntary inpatient history possessed a slightly higher odds ratio (OR = 2.337, 95% CI: 1.485–3.677) than voluntary inpatient (OR = 1.980, 95% CI: 1.216–3.226). The inpatient history was not significantly associated with F1 diagnosis [$\chi_{\left(1\right)}^{2}$ = 0.113, *p* = 0.803]. To summarize, risk factors for overall problematic behaviors included F1 diagnosis and inpatient history prior to MTSA-based treatment.

## Discussion

### General Results

The present study collected data from 441 clinical offenders, or approximately 56% of the 779 patients receiving MTSA outpatient treatment from 15 July 2005 to 31 December 2009, with cooperation from 158 nationwide outpatient institutions in Japan. The main aim of this study was to clarify risk factors for overall problematic behaviors, using demographic, psychiatric, forensic, clinical treatment, and social service information. Cascade categorical analyses were applied to elucidate factors related to patients’ inclusion in MTSA-based treatment, and their commitment of problematic behaviors. Among 443 patients, 189 (43.0%) committed problematic behaviors, and 293 problematic behaviors were counted, which included dominant problematic behaviors, such as medical non-compliance (25%), violent behaviors (24%), substance abuse (10%), and self-harming and suicide (7%). Risk factors for overall problematic behaviors included a positive F1 diagnosis (OR = 3.211) and inpatient history (OR = 1.502), which were not significantly related to each other. Especially, F1 diagnosis was widely correlated with other demographic, forensic, and clinical treatment-related profiles. These findings indicate that reduction of overall problematic behaviors under MTSA-based outpatient treatment advances under protective attention to patients with past substance use problems and/or past inpatient history likely for a more severe psychiatric symptom. Immediately below, we will first argue about the risk factors for problematic behaviors, and subsequently summarize patient profiles under MTSA-based outpatient treatment.

### Risk Factors for Problematic Behaviors

Risk factors for overall problematic behaviors in the course of MTSA-based outpatient treatment included F1 diagnosis (OR = 3.211) and inpatient history before treatment (OR = 1.502). These factors were not significantly correlated with each other.

An F1 diagnosis was a risk factor for overall problematic behaviors. As has been widely reported in previous studies, alcohol and/or substance abuse are generally related to problematic behaviors [for a review, see Ref. (18)]. During a 30-year birth cohort study in New Zealand, for example, people (17–30 years) with severe alcohol and substance abuse symptoms, compared to those without these symptoms, possessed about 12 times higher risk of committing violent behaviors (19). Concerning substance abuse, a Norwegian study (13–30 years; *n* = 1,353) also reported that abnormal substance or cannabis use was a risk factor for overall crimes, and compared to non-use, had three times higher risk for crimes in adolescents (15–20 years) (20). Because F1 symptoms are related not only to violence-related problematic behaviors, but also medical non-compliance to, for example, substance abuse interventions (11), it may be important to intensively attend to patients’ substance use as well as overall medical non-compliance.

Another risk factor was inpatient history before MTSA-based treatment. Individuals with inpatient history, compared to those without, had 1.5 times the risk for problematic behaviors. This suggests that clinical offenders previously required enhanced observation or interventions (21), likely because their psychiatric symptoms became acute or they showed a risk of committing problematic behaviors. This assumption may be supported by the additional analyses, summarized in Figure 2D. We examined inpatient histories and whether patients received care voluntarily or involuntarily (medical care or involuntary admission). Both voluntary and involuntary inpatient histories were risk-enhancing factors for problematic behaviors. Additionally, involuntary inpatient history showed a slightly higher odds ratio (OR = 2.337) than did voluntary inpatient history (OR = 1.980). Patients with relatively severe psychiatric symptoms, therefore, tend to possess inpatient histories before MTSA-based treatment, and in particular, patients with involuntary inpatient history may be exposed to high risks of committing future problematic behaviors. These findings suggest that patients who were previously involuntary inpatients should receive more intense MTSA-based treatment monitoring.

### Predominant Profiles of Patients under MTSA-Based Outpatient Treatment

Predominant profiles of patients under MTSA-based outpatient treatment included various demographic, psychiatric, forensic, and clinical treatment factors. The patient population was predominantly male (about 70%). Such male dominance in clinical offenders was consistently observed in previous Japanese studies, which comprised partially overlapping patients undergoing hospitalization for assessment (16) and under MTSA-based inpatient treatment (22, 23). Additionally, a study from the United States, recruiting patients under involuntary inpatient treatment who committed serious violence, reported that male patients tended to more often commit violent acts several months before treatment admission than female patients (24). Another general adolescent violence study randomly selected 1,046 students from schools in Dubai, the United Arab Emirates, and examined relevant socio-demographic factors (25). This study also revealed that male adolescents possessed 1.6 times higher risk for violence than females. Furthermore, large cohort studies, those supported by the National Institute of Justice and the Centers for Disease Control and Prevention in the United States, collected assault data from 16,000 individuals (8,000 women and 8,000 men) (26). From the perspective of victims, this survey reported that about 22% of women, compared to 8% of men, were physically assaulted by their intimate partners, which also demonstrates gender asymmetry in violent behaviors. Although these results tend to support male dominance in problematic violent behaviors, on the other hand, we should be careful not to overestimate these findings, and also take into consideration social or clinical situations, where female patients may dominantly commit violence or problematic behaviors (27, 28).

Lower school history (≤high school) was common among patients. This may be due to a larger proportion of F2 diagnoses (77%, *n* = 340), which are the most common diagnosis among younger populations, in patients with lower education (junior high or less: *n* = 131; high school: *n* = 146) compared to those with higher education (university: *n* = 63). Because higher education (university) was a risk factor (OR = 4.384) for F3 diagnosis, differences in education history may likely be sensitive to psychiatric diagnosis distinctions.

Diagnoses of F1, F2, and F3 were varied in proportions across patients (F1: 6%; F2: 77%; F3: 43%). The MTSA mainly treats clinical offenders with F2 diagnoses because of their treatability to overcome several obstacles, such as the timing of treatment interventions and clinical conditions for treatment (29). Therefore, it is reasonable that F2 diagnoses were most common among patients under MTSA-based treatments.

On the other hand, F1 diagnosis was not only the risk factor for overall problematic behaviors but was also related to male gender (OR = 3.419) and past correctional admission (OR = 13.759). Hence, substance use disorder, when correlated with other risk factors, may be persistently related to criminal behaviors subject to MTSA-based treatments. Because F1 diagnosis was inversely related to outpatient history before MTSA-based treatment and direct outpatient treatment, patients with an F1 diagnosis may be a population hard to recruit into outpatient treatment in general.

F3 diagnosis (mood disorders) was inversely associated with male gender (OR = 0.36), indicating that F3 was proportionally dominant among female patients. This also confirms a diagnosis profile of patients found under MTSA treatment from a single facility in our country (22); that is, female patients (28%) are more often diagnosed as F3 than male patients (4%). F3 diagnosis was also increasingly related to homicide in both present and previous studies. Nagata et al. (22) reported that female patients (56%) committed homicide (infanticide or “shinju (double suicide)”) more frequently than male patients (31%). To summarize, F3 diagnosis is likely a robust risk factor for MTSA-based treatment inclusion due to homicide, in particular for female patients.

Dominant crime types included sexual and physical assaults (39%), arson (34%), and homicide (25%). Although familiar persons (62%) were more often victims than strangers (38%), crime types were related to victim types. Homicide and arson included familiar victims such as family members, partners, or acquaintances (80–90%) more often than strangers, while assaults included strangers (64%) more often than familiar persons (36%). It has been reported that with respect to clinical offenders with mental disorders, victim types are related to criminal types. In Sweden cohort studies, for example, about 80% of victims were familiar to clinical offenders with schizophrenia who committed homicide (30, 31). These findings indicate that interaction between crime type and psychiatric disorder affects victim type.

Almost all patients (93%) did not have past experiences in correctional institutions. MTSA-based treatment aims to promote medical treatment and reintegration into local communities among patients committing serious crimes when they were in a state of insanity or diminished responsibility caused by mental disorders (32). Thus, individuals with personality-related or conduct disorders tend to be excluded. This may explain the lack of correctional history among patients.

About 80% of patients had been linked in some way to psychiatric outpatient treatment before their crime, and about half had undergone involuntary hospitalization for psychiatric treatment or hospitalization for medical care and protection on the grounds that they posed a danger to themselves or others. Past clinical treatment profiles suggest that patients with past treatment history, compared to those without treatment history, tend to possess relatively severe psychiatric disorders, and to be more often included under MTSA-based treatment. Although this study did not analyze clinical treatment data at the time of the commitment of the offense, at least 30% of patients had been clinically treated at the time of committing the offense. That is, monitoring the remaining 70% of the patients’ interrupted or non-existent treatment may be effective for preventing future crimes and also be necessary to ensure continuity of outpatient treatment. It is also likely effective to suppress problematic behaviors under the MTSA-based treatment to specify kinds of treatments and clinical supports required by 30% of patients who committed offenses even during clinical treatment before MTSA-based treatments. Based on the accumulating MTSA-based psychiatric treatment histories in our and cooperating facilities, not only pharmacotherapy, but also various programs for rehabilitation, such as disease education and social skills training, should be combined to provide practical life support. At the same time, it may be efficacious to educate patients’ family members about disorders while supporting the overall lifestyle and medical treatment of patients after or before MTSA-based treatment decisions. This education may protect against offenses during MTSA-based treatment and also promote the treatment success.

## Conclusion

The nationwide data collection and analyses of patients receiving MTSA-based outpatient treatments likely provide meaningful and substantial suggestions regarding the creation of a crisis plan from the viewpoint of risk management. The present findings imply that reduction of overall problematic behaviors under MTSA-based outpatient treatment is promoted with care for patients with substance use problems and/or past inpatient history. The highest risk factor of F1 diagnosis also suggests that biological aspects of abnormal neural function and representation caused by substance use should also be taken into consideration as a risk assessment item for MTSA-based treatment for patients’ social reintegration. Based on continuous collection and analyses of additional information in the future, a more comprehensive and practically useful clinical guideline could be created for the MTSA system. This plan would aid in the successful reintegration of patients, and could provide useful information to national and potentially international forensic and general psychiatric institutions. Hence, this study should be replicated for data accumulated during the new study period.

## Author Contributions

KA, KN, TN, and TO conceived and designed the present study. KA and KN collected data. KA, KN, and TS analyzed the data. KA, TS, KN, TN, and TO interpreted the data. KA and TS drafted and revised the manuscript. KA, TN, and TO supervised the study. All authors approved the final manuscript.

## Conflict of Interest Statement

The authors declare that the research was conducted in the absence of any commercial or financial relationships that could be construed as a potential conflict of interest.
